# Emergently Alteration of Procedural Strategy During Transcatheter Aortic Valve Replacement to Prevent Coronary Occlusion: A Case Report

**DOI:** 10.3389/fcvm.2022.931595

**Published:** 2022-07-27

**Authors:** Hanyi Dai, Dao Zhou, Jiaqi Fan, Lihan Wang, Abuduwufuer Yidilisi, Gangjie Zhu, Jubo Jiang, Huajun Li, Xianbao Liu, Jian’an Wang

**Affiliations:** ^1^Internal Medicine, Zhejiang University School of Medicine, Hangzhou, China; ^2^Department of Cardiology, The Second Affiliated Hospital Zhejiang University School of Medicine, Hangzhou, China

**Keywords:** aortic valve stenosis, transcatheter aortic valve replacement, emergent, pre-dilatation, procedural strategy

## Abstract

**Background:**

Coronary occlusion is an uncommon but fatal complication of transcatheter aortic valve replacement (TAVR) with a poor prognosis.

**Case Presentation:**

A patient with symptomatic severe bicuspid aortic valve stenosis was admitted to a high-volume center specializing in transfemoral TAVR with self-expanding valves. No anatomical risk factors of coronary occlusion were identified on pre-procedural computed tomography analysis. The patient was scheduled for a transfemoral TAVR with a self-expanding valve. Balloon pre-dilatation prior to prosthesis implantation was routinely used for assessing the supra-annular structure and assessing the risk of coronary occlusion. Immediately after the tubular balloon inflation, fluoroscopy revealed that the right coronary artery was not visible, and the flow in the left coronary artery was reduced. The patient would be at high-risk of coronary occlusion if a long stent self-expanding valve was implanted. Therefore, our heart team decided to suspend the ongoing procedure. A transapical TAVR with a 23 mm J-valve was performed 3 days later. The prosthesis was deployed at a proper position without blocking the coronary ostia and the final fluoroscopy showed normal flow in bilateral coronary arteries with the same filling as preoperatively.

**Discussion:**

Our successful case highlights the importance of a comprehensive assessment of coronary risk and a thorough understanding of the TAVR procedure for the heart team. A short-stent prosthesis is feasible for patients at high risk of coronary occlusion. Most importantly TAVR should be called off even if the catheter has been introduced when an extremely high risk of coronary obstruction is identified during the procedure and no solution can be found.

## Introduction

Transcatheter aortic valve replacement (TAVR) has currently revolutionized as the guideline-recommended therapy for the elderly with severe aortic valve stenosis (1). Coronary occlusion is an uncommon but potentially fatal complication with an incidence of less than 1% (2). Coronary occlusion would result in unplanned surgical or interventional treatment, myocardial infarction, and even death (2, 3). As TAVR has advanced notably in recent years, the evaluation and prevention of coronary occlusion are still the main concerns.

## Case Presentation

**FIGURE 1:**
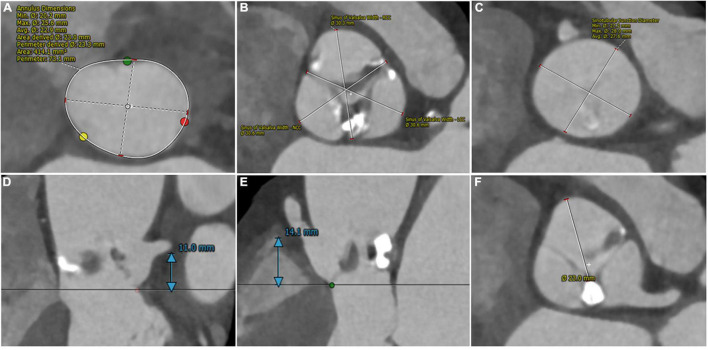
Pre-procedural computed tomography assessment of aortic root. **(A)** Annular perimeter: 73.1 mm and annular area: 414.1 mm^2^. **(B)** Left-, right- and non-sinuses of Valsalva dimensions: 31.6 mm * 30.7 mm * 30.7 mm. **(C)** Average sinotubular junction diameter: 27.6 mm. **(D,E)** The height of coronary arteries: 11 mm (left) and 14.1 mm (right). **(F)** The distance from the edge of the calcified nodule to right coronary ostium: 23 mm.

A 75-year-old woman diagnosed with severe aortic valve stenosis was admitted to our hospital for progressive exertional dyspnea in the last 3 years ago with New York Heart Association (NYHA) Class III functional status. She had clinical comorbidities of Parkinson’s disease and pulmonary space-occupying lesions. Cardiac auscultation detected a 3/6 mid-systolic murmur with maximum intensity in the area of the right upper sternal border. Echocardiography demonstrated severe calcified aortic valve stenosis with mild-to-moderate regurgitation (valve area = 0.85 cm^2^; peak velocity = 4.23 m/s; and mean transvalvular gradient = 42 mmHg). Left ventricular ejection fraction (LVEF) estimated by the two-dimensional methods was 61.4%. Multi-detected CT (MDCT) identified that the aortic valve was bicuspid aortic valve type 1 and a raphe with a bulky calcification between the left and non-coronary sinuses (4, 5). Further anatomical measurements on MDCT included an annular perimeter of 73.1 mm, an annular area of 414.1 mm^2^, and the left main coronary artery (LMA) ostium height of 11 mm and right coronary artery (RCA) of 14.1 mm (Figures 1A,B,D,E). The dimensions of left-, right-, and non-coronary sinuses were 31.6, 30.7, and 30.7 mm, respectively (Figure 1B). The average dimension and the height of the sinotubular junction were 27.6 and 16 mm (Figure 1C). Measured on the plane of the right coronary ostium, the distance of the edge of the calcified nodule to right coronary ostium was 23 mm (Figure 1F). The average internal diameters of the right femoral-, external iliac-, and common iliac arteries were 6.7, 6.5, and 7.3 mm, respectively. The patient was deemed at intermediate surgical risk with the Society of Thoracic Surgeons score of 5.13% and was recommended for surgical aortic valve replacement (SAVR). However, the patients refused SAVR and were determined to undergo minimally invasive TAVR.

**FIGURE 2:**
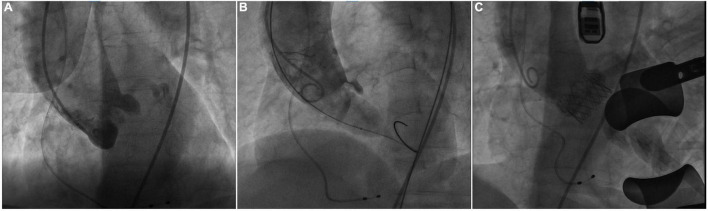
Intraoperative fluoroscopy. **(A)** Fluoroscopy showed bilateral patent coronary arteries before balloon pre-dilatation. **(B)** The right coronary artery was invisible and reduced blood flow in the left coronary artery during the balloon dilatation. **(C)** Fluoroscopy showed the patency of bilateral coronary arteries after J-valve deployment.

The procedure was performed in our hybrid operating room for TAVR as previously described (4). At the beginning of the TAVR procedure, bilateral coronary arteries were patent under intraoperative fluoroscopy (Figure 2A). Balloon pre-dilatation was routinely performed to assess the supra-annular structure and evaluate the risk of coronary occlusion using a 23 mm × 40 mm Z-Med™ balloon based on the annulus perimeter derived diameter (6). Unexpectedly, the right coronary artery was invisible and the blood flow reduced in the LMA, immediately after balloon dilatation (Figure 2B), indicating RCA completely occluded and LMA partially occluded by the native left coronary leaflet. The patient’s blood-oxygen saturation dropped sharply from 100 to 72%. Intraoperative transthoracic echocardiography revealed a transvalvular gradient of 20 mmHg following balloon deflation. Therefore, the multidisciplinary team decided to suspend the procedure. A careful and comprehensive reassessment of the patient’s anatomy was performed and the transapical TAVR with J-valve (JieCheng Medical Technology Co., Ltd., Suzhou, China) was planned for safety consideration.

Subsequently, transapical TAVR with J-valve was performed under general anesthesia 3 days later. A 23-mm J-Valve system was introduced and repeatedly adjusted to ensure the three graspers were properly placed in the left-, right-, and non-coronary sinuses and accurately surround the native leaflets. The prosthetic valve was gradually released after positioning at 1 cm below the aortic annulus under rapid ventricular pacing. The procedure was uneventful and the final fluoroscopy showed the proper position of the prosthetic valve and the patency of bilateral coronary arteries (Figure 2C). The final echocardiography showed mild perivalvular leakage and the residual transvalvular gradient was 3 mmHg. The patient was discharged without obvious symptoms of heart failure and could carry out daily activities with ease after discharge. At 30 days follow-up, she had an NYHA class II functional status and echocardiography showed normal aortic valve function with a transvalvular mean gradient of 5 mmHg.

## Discussion

To the best of our knowledge, this is the first case of suspending an ongoing procedure for an elective procedure with another type of valve when faced with an extremely high risk of coronary occlusion during the procedure.

Coronary occlusion is defined as complete or partial obstruction of coronary ostia during or after the procedure by native aortic leaflets, the prosthesis valve, calcified nodules, etc. It is a devastating complication that compromises survival and affects prognosis (2, 3). Preprocedural MDCT assessment is an optimal assessment technique to identify anatomical risk factors of coronary occlusion (7). Redundant leaflet, heavy calcification, coronary ostia height < 10 mm, sinus of Valsalva dimensions < 30 mm, and leaflet length to coronary sinus height ratio greater than 1 are anatomic predictors of coronary occlusion (2, 8). A comprehensive review of our case especially pre-procedural MDCT analysis could not identify any of the typical anatomical risk factors for coronary occlusion. However, the length of the left coronary cusp was greater than the height of the left coronary ostium, and the bulky calcium nodule located on the L-N raphe may force the valve displacement to hinder the contralateral right coronary ostium (Figure 1). Thus, the heart team considered the patient’s coronary occlusion risk cannot be ignored but not at high risk. Balloon pre-dilatation was considered for evaluating the possibility of coronary compromise during the procedure (3). Luckily, the extremely high risk of coronary occlusion was detected by balloon pre-dilatation.

However, we thought there existed no great backup methods for this patient during that procedure. BASILICA technique allows flowing into the coronary ostia by intentionally splitting native or bioprosthetic leaflets (9). However, this technique is targeted at coronary occlusion caused by valve displacement and it may not be suitable for our case. The chimney stenting implantation technique is a bailout technique used to treat coronary occlusion during TAVR. The interaction between the prosthetic valve edge and the chimney stent may lead to deformation of the coronary stent and prosthesis. Long-term effects on stent thrombosis and valve durability remain unknown. Besides, this technique is mainly targeted at LMA. Only 6 patients in the International Chimney Registry received bilateral chimney stents contemporarily and the prognosis is unclear (10). The several techniques mentioned above might not be our best option to avoid coronary occlusion. Severe aortic stenosis was temporarily relieved through balloon pre-dilatation. Thus, we called off the procedure to avoid harm to the patient, comprehensively re-assessed the procedural strategy, and decided to replace it with another short-stent valve.

**FIGURE 3:**
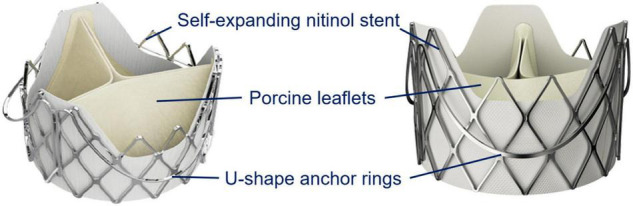
The J-Valve system design. The J-Valve aortic system consists of a self-expanding nitinol stent surrounded by three U-shaped anchor rings, with porcine aortic valve leaflets sutured inside. This draft was provided by the company JieCheng Medical Technology Co., Ltd., Suzhou, China.

The J-Valve system is a self-expanding prosthesis with a transapical delivery sheath for both aortic stenosis and aortic regurgitation (Figure 3). The unique structure with three anatomical orientated U-shaped anchors, short stent frame and even lower profile in the regions of the coronary ostia is effective for positioning stabilizing, and avoiding coronary occlusion (11). Patients with bicuspid aortic valve without raphe (Type 0) are considered to be the contraindication for the J-valve system and are always excluded from well-designed studies (12, 13). Type 1 represents three coronary cusps with one fusion raphe between two adjacent cusps. Tung et al. reported that the 1- year major adverse cardiovascular events-free survival was similar between tricuspid vs. bicuspid aortic valve (log-rank *p* = 0.25), which indicated the safety and efficacy of the J-Valve implantation in patients with bicuspid morphology (12). Thus, our heart valve team decided to discontinue the current procedure and reschedule another procedure with J-Valve to avoid fatal coronary occlusion. As the result, we avoided coronary occlusion which may occur after long-stent valve implantation and obtained satisfactory results. Our successful case suggests that the patients’ safety is paramount. It is feasible to suspend the procedure when extremely high operative risk is detected during the procedure, even if the catheter has been introduced. In this situation, reassessing the patient’s anatomic structure comprehensively, modifying the procedural strategy, and even recommending patients to undergo the procedure again should be considered.

## Conclusion

Coronary artery occlusion is a fatal complication with high mortality. Our case emphasizes the importance of a comprehensive assessment of the interaction between the coronary ostia and surrounding structures for TAVR candidates and the feasibility of emergently restructuring strategy intraoperatively. A short stent prosthesis is feasible for patients with high coronary occlusion risk. More importantly, ceasing the procedure which would do harm to patients was necessary, even if the catheter has been deployed into the patient’s body.

## Data Availability Statement

The original contributions presented in this study are included in the article/supplementary material, further inquiries can be directed to the corresponding author/s.

## Ethics Statement

The studies involving human participants were reviewed and approved by Institutional Review Board of The Second Affiliated Hospital Zhejiang University School of Medicine. The patients/participants provided their written informed consent to participate in this study. Written informed consent was obtained from the individual(s) for the publication of any potentially identifiable images or data included in this article.

## Author Contributions

HD and DZ contributed to composing the manuscript. JF collected the patient’s data and draw figures. LW, AY, GZ, JJ, and HL revised the manuscript. XL and JW analyzed and explained the steps of procedures, as well as conceived and revised the manuscript. All authors contributed to the article and approved the submitted version.

## Conflict of Interest

The authors declare that the research was conducted in the absence of any commercial or financial relationships that could be construed as a potential conflict of interest.

## Publisher’s Note

All claims expressed in this article are solely those of the authors and do not necessarily represent those of their affiliated organizations, or those of the publisher, the editors and the reviewers. Any product that may be evaluated in this article, or claim that may be made by its manufacturer, is not guaranteed or endorsed by the publisher.

## Abbreviations

TAVR: transcatheter aortic valve replacement
NYHA: New York Heart Association
LVEF: Left ventricular ejection fraction
MDCT: multi-detected computed tomography
LMA: left main coronary artery
RCA: right coronary artery
SAVR: surgical aortic valve replacement.
